# Immune regulation by protein ubiquitination: roles of the E3 ligases VHL and Itch

**DOI:** 10.1007/s13238-018-0586-8

**Published:** 2018-11-09

**Authors:** Daisuke Aki, Qian Li, Hui Li, Yun-Cai Liu, Jee Ho Lee

**Affiliations:** 1 Institute for Immunology, Tsinghua-Peking Center for Life Sciences , School of Medicine, Tsinghua University, Beijing, 100084 China; 2 La Jolla Institute for Allergy and Immunology, La Jolla, CA 92037 USA

**Keywords:** ubiquitin, E3 ligase, VHL, HIF, Itch, WWP2, Cbl-b, inflammation, autoimmunity

## Abstract

Protein ubiquitination is an important means of post-translational modification which plays an essential role in the regulation of various aspects of leukocyte development and function. The specificity of ubiquitin tagging to a protein substrate is determined by E3 ubiquitin ligases via defined E3-substrate interactions. In this review, we will focus on two E3 ligases, VHL and Itch, to discuss the latest progress in understanding their roles in the differentiation and function of CD4^+^ T helper cell subsets, the stability of regulatory T cells, effector function of CD8^+^ T cells, as well as the development and maturation of innate lymphoid cells. The biological implications of these E3 ubiquitin ligases will be highlighted in the context of normal and dysregulated immune responses including the control of homeostasis, inflammation, auto-immune responses and anti-tumor immunity. Further elucidation of the ubiquitin system in immune cells will help in the design of new therapeutic interventions for human immunological diseases and cancer.

## Introduction

Our immune system is tightly regulated to protect against invading pathogens, and at the same time to prevent from self-destruction. The immune responses are dependent on numerous cellular signaling pathways and their cross-interactions in immune cells. To achieve the selectivity and diversity of each pathway, post-translational modification plays an important role in regulating the function of key proteins during signal transduction cascades. The tagging of a small 76 amino acid polypeptide to a protein, known as protein ubiquitination, is an essential post-translational modification. Protein ubiquitination is carried out via a sequential enzymatic cascade by E1 ubiquitin-activating enzymes, E2 ubiquitin-conjugating enzymes and E3 ubiquitin ligases (Komander, 2009). The E3 ligases determine the specificity by selectively recruiting particular protein substrates via defined protein-protein interactions. The E3 ligases can be classified into two classes. One is the RING (really interesting new gene)-type E3 ligases and the other is the HECT (homologous to the E6AP carboxyl terminus)-type E3 ligases, depending on the structure of specific E2 binding domains. The RING-type E3 ligase utilizes the RING domains to recruit and activate the ubiquitin-conjugated E2 enzymes to transfer ubiquitin to the substrate, whereas in the HECT-type E3 ligase, the HECT domain accepts ubiquitin on its cysteine residue from the E2 enzyme, and then directly transfers the ubiquitin to the specific substrate (Lorick et al., 1999; Metzger et al., 2012).

Protein ubiquitination plays an important role in diverse cellular responses such as cell cycle, signal transduction pathway, transcriptional regulation, DNA repair and apoptosis (Gilberto and Peter, 2017). In the immune system, ubiquitin-tagged modification has been implicated in both innate and adaptive immune responses (Liu, 2004). Particularly, E3 ligases have been shown to regulate the development, differentiation and activation of various leukocytes, and their biological functions. In this review, we will focus on two E3 ligases, the von Hippel-Lindau (VHL) and Itch, to discuss the latest new findings in the cellular and molecular mechanisms by which they control immune homeostasis under steady state and how the loss of them causes immune dysregulation under pathological conditions.

### The E3 ligase VHL

**Figure 1:**
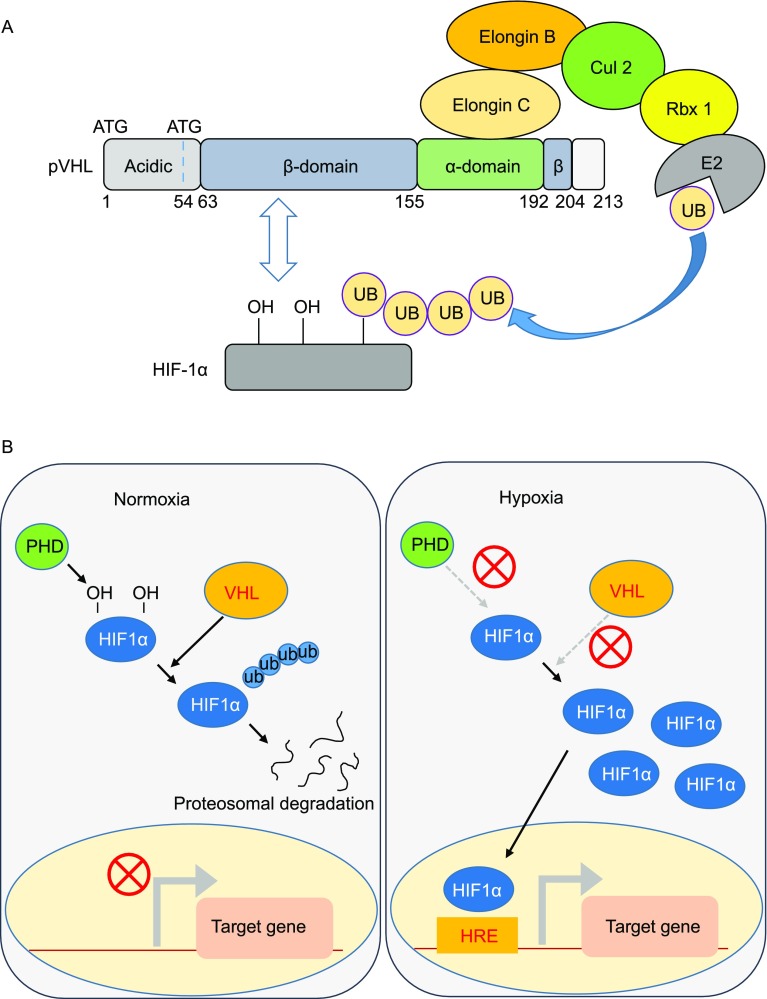
**VHL-HIF pathway**. (A) Schematic structure of VHL protein shows that the β-domain binds to hydroxylated HIF-1α, whereas the α-domain is for the binding of key components in the E3 ligase complexes. (B) Under normoxic conditions, PHD hydroxylates HIF-1α, which is recognized and ubiquitinylated by VHL, followed by degradation through the proteasome pathway. During hypoxia, non-hydroxylated HIF-1α is able to escape from ubiquitination by VHL, and thus accumulated HIF-1α translocates into the nucleus to turn on the transcription of its target genes

In humans, inactivation or mutation of the von Hippel-Lindau (VHL) gene results in tumor development in multiple tissues including the kidneys, pancreas, retina, uterus, central nervous system and the adrenal gland (Kaelin and Maher, 1998; Kim and Kaelin, 2004). The VHL E3 ligase complex is composed of elongin B, elongin C, cullin 2 and ring box protein 1 (Rbx1) (Kamura et al., 1999; Stebbins et al., 1999) (Fig. 1). Hypoxia-inducible factor-1α (HIF-1α) is well-known as a substrate of the VHL E3 ligase complex and is a master transcription factor that regulates the gene expression for glucose metabolism and angiogenesis at low oxygen levels (Zhang et al., 2007). HIF-1α is recognized as an oxygen sensor and thus plays an important role in adaptations to hypoxia. Although most mammalian cells show the constitutive expression of HIF-1α mRNA (Kallio et al., 1997), HIF-1α protein is maintained at a low level under normoxia, due to its hydroxylation by prolyl hydroxylase domain (PHD) enzymes, the recognition by VHL, and the subsequent degradation by proteasome (Maxwell et al., 1999; Ivan et al., 2001). Under hypoxic conditions, PHD activity is reduced, which prevents the binding of HIF-1α to VHL, and thus leads to the accumulation of HIF-1α thereby promoting the transcriptional activation of various genes. This biochemically well-defined oxygen-sensing VHL-HIF pathway, whose discovery leads to the 2016 Lasker award, plays an important role in the regulation of immune cells.

### VHL in T cells

During T cell development, hematopoietic progenitor cells migrate from bone marrow into the thymus, a primary lymphoid organ known to be under hypoxic conditions (Hale et al., 2002). The specific deletion of VHL has an impact on thymocyte development by inducing cell death through increased caspase activity (Biju et al., 2004). In addition, HIF-1α induced by VHL deficiency regulates Ca^2+^ response upon T cell receptor (TCR) stimulation during thymocyte development (Neumann et al., 2005). Moreover, hypoxia can affect T cell differentiation in the peripheral tissues. Recent studies have demonstrated that HIF-1α is a key transcription factor for T helper cell type 17 (Th17) and regulatory T cell (Treg) differentiation (Dang et al., 2011; Shi et al., 2011). HIF-1α is able to promote the gene expression of RORγt, a master transcription factor for Th17 cells, while suppressing Treg generation by downregulating Foxp3 (Dang et al., 2011). The HIF-1α-dependent glycolytic pathway is shown to regulate the balance of Th17 and Treg polarization by the utilization of a glycolytic inhibitor (Shi et al., 2011). On the contrary, other groups have reported that the HIF-1α pathway promotes Foxp3 expression (Ben-Shoshan et al., 2008; Clambey et al., 2012). Thus, it remains unclear how the VHL/HIF pathway regulates Treg development and/or function.

Our own study revealed that VHL controls Treg stability and function by regulating HIF-1α by utilization of *Vhl*^fl/fl^ *Foxp3*^cre^ conditional knockout mice (Lee et al., 2015). In VHL-deficient Treg cells, HIF-1α is able to induce the production of interferon-γ (IFN-γ) by transcriptional activation by binding onto the *Ifng* gene locus. This further promotes Th1-domininant inflammation in conditional VHL knockout mice. The induction of IFN-γ by the VHL/HIF pathway is also required for glycolytic metabolism. Furthermore, a recent study reported that IFN-γ-producing Treg cells regulated by neuropilin-1 promote anti-tumor immunity through hypoxia that controls Treg fragility via HIF-1α (Overacre-Delgoffe et al., 2017). Another recent study also showed that PHD protein restrains T cell-intrinsic anti-tumor immunity by the regulation of Th1 immune response (Clever et al., 2016). These findings suggest that the PHD/HIF/VHL pathway is responsible for IFN-γ expression in Th1 immunity during tumor development as well as autoimmunity.

In addition, the VHL-HIF pathway is also involved in CD8 T cell development. T cell-specific VHL deletion in mice shows lethal pathology during persistent LCMV infection (Doedens et al., 2013). This is due to the fact that VHL deficiency-mediated HIF-1α and HIF-2α enhance the function of cytotoxic CD8 T lymphocytes by promoting the expression of key transcription factors, effectors and costimulatory/inhibitory molecules. On the other hand, VHL-deficient cytotoxic T cells are more effective in preventing tumor growth with their enhanced cytotoxicity. Moreover, further study demonstrates that stable HIF-1α by VHL deletion does not affect the differentiation of long-lived memory CD8 T cells although VHL deficiency sustains glycolytic metabolism. However, glycolytic metabolism promotes the differentiation of T effector memory cells, but not T central memory CD8^+^ cells (Phan et al., 2016). This indicates that metabolic control by the VHL/HIF axis will be critical to generate protective CD8^+^ memory T cell against infections.

### VHL in B cells

Bone marrow is known as a site with limited oxygen supply (Eliasson and Jonsson, 2010), and thus, it is likely that B cell development is regulated by hypoxia. Indeed, the absence of HIF-1α leads to impaired B cell development in the bone marrow due to defective proliferation of HIF-1α-deficient B cells. It has been noted that activated B cells produce interleukin-10 (IL-10) to inhibit autoimmunity by suppressing pathogenic T cells (Nakashima et al., 2010; Matsumoto et al., 2014) and HIF-1α deficiency results in an increased B1-like cell population which further causes autoimmunity (Kojima et al., 2002). HIF-mediated glycolysis suppresses the expression of IL-10 from CD1d^hi^CD5^+^ B cells. HIF-1α and STAT3 cooperatively regulate IL-10 expression. Compared to WT control mice, HIF-1α-deficient mice are more susceptible to autoimmune diseases such as arthritis and experimental autoimmune encephalomyelitis (Evans et al., 2007; Matsushita et al., 2008). HIF-1α knockout B cells restrain Th1 and Th17 differentiation through IL-10 production (Meng et al., 2018). These findings support that the regulation of hypoxia is critical for B cell function in preventing autoimmunity.

More interestingly, hypoxia in the germinal center (GC) regulates antibody production by B cells. HIF-1α limits the proliferation, survival and isotype switching (Abbott et al., 2016). Low oxygen tension or B cell-specific VHL depletion restrains mTORC1 activity, thus inhibiting B cell activation, which is restored by HIF-1α deletion, indicating that the VHL/HIF pathway plays an important role in antibody production in the GC during immune responses (Cho et al., 2016). These findings suggest that oxygen sensing through the VHL-HIF pathway is essential to regulate humoral immunity in the lymphoid tissues.

### VHL in myeloid cells

The VHL-HIF axis also has an impact on innate immunity (Rius et al., 2008; Palazon et al., 2014). In fact, hypoxia is able to promote the suppressive function of myeloid-derived suppressor cells with upregulated arginase-1 and iNOS expression, which is dependent on HIF-1α accumulation (Corzo et al., 2010). Another study demonstrates that low oxygen concentration suppresses neutrophil apoptosis through HIF-1α-dependent NF-κB signaling (Walmsley et al., 2005). Thus, these findings support that the VHL/HIF pathway plays an important role in the development and function of myeloid cells. A more recent study utilized conditional VHL knockout mice by crossing *Vhl* floxed and *CD11c*-cre mice (Izquierdo et al., 2018). Interestingly, alveolar macrophages (AMs) show immature phenotypes due to VHL deficiency with reduced self-renewal capacity, indicating that VHL is required for maturation of AMs. In addition, the surfactant handling activity by AMs is also decreased by VHL deficiency, indicating VHL is responsible for the function of AMs to clear pulmonary surfactant. Thus, these data suggest that the VHL/HIF axis is essential in terminal differentiation, self-renewal and function of AMs.

### VHL in innate lymphoid cells

Innate lymphoid cells (ILCs) are a recently defined subset of lymphocytes that reside in peripheral tissues and are particularly abundant at barrier surfaces. ILCs are functionally divided into three subsets: group 1 ILCs including natural killer cells and IFN-γ producing ILC1s that depend on T-bet for their development; group 2 ILCs that produce type 2 inflammatory cytokines and require GATA3 and RORγ; and group 3 ILCs that produce IL-17A and/or IL-22 and depend on RORγt (Spits et al., 2013). They originate from common lymphoid progenitors that commit to ILC precursors expressing the transcription factor PLZF, which have the capacity to differentiate into mature ILCs (Constantinides et al., 2014). However, whether the VHL-HIF pathway is involved in the regulation of ILCs remains elusive.

To address this issue, we generated *Vhl*^fl/fl^ *Plzf*^cre^ mice to selectively deplete the VHL gene in ILC precursors and demonstrated that VHL plays a pivotal and selective role in the development and function of ILC2s through the HIF-1α pathway (Li et al., 2018). ILC2s can respond to alarmin cytokines such as IL-33, IL-25 and TSLP to produce type 2 cytokines, thus playing a key role in allergic diseases, anti-helminth infection and metabolic homeostasis (Ebbo et al., 2017). Conditional deletion of VHL in innate lymphoid precursors minimally affected early-stage bone marrow ILC2s, but caused a selective and intrinsic decrease in mature ILC2 numbers in peripheral non-lymphoid tissues such as the lung, intestine and lipid tissues, resulting in reduced type 2 immune responses. As expected, VHL deficiency caused the accumulation of HIF1α, which was rectified by HIF-1α ablation. In addition, IL-33 receptor ST2 expression was attenuated in VHL-deficient ILC2s. Further mechanistic studies showed that the HIF-1α-driven metabolic shift altered the epigenetic modification on the ST2 gene and thus inhibited IL-33-induced ILC2 development.

Taken together, the VHL/HIF pathway is critical for the development and function of various immune cells (Table 1). Since hypoxia can affect diverse cells simultaneously in a tissue environment, further studies will investigate the mutual interplay between immune cells to understand the detailed mechanism for their function during inflammatory immune responses or tumor development.

**Table 1:** The major phenotype of VHL conditional knockout mice.

Target cells	cKO mice	Phenotype	References
Thymocytes	*Vhl* ^fl/fl^ *Lck-cre*	- increases cell death and caspase activity - reduces TCR-mediated Ca^2+^ signaling	(Biju et al., 2004) (Neumann et al., 2005)
CD4 Treg cell	*Vhl* ^fl/fl^ *Foxp3-cre*	- loss of Treg suppressive function - IFN-γ-mediated Th1 dominant inflammation	(Lee et al., 2015)
CD8 cell	*Vhl* ^fl/fl^ *dLck-cre*	- mortality in persistent viral infection - promotes cytotoxic killing - enhances T_em_ differentiation via glycolytic metabolism	(Doedens et al., 2013) (Phan et al., 2016)
B cell	Transfer model with B cells from *Vhl*^fl/f*l*^ *ER*^*T2*^*-cre*	- reduced GC function - defective in clonal expansion, Ab production	(Cho et al., 2016)
Macrophage	*Vhl* ^fl/fl^ *Cd11c-cre*	- defective in alveolar macrophage maturation, self-renewal, function	(Izquierdo et al., 2018)
Innate lymphoid cell	*Vhl* ^fl/fl^ *Plzf-cre*	- defective in ILC2 differentiation - downregulated ST2 expression by metabolic shift	(Li et al., 2018)

### The E3 ligase Itch

**Figure 2:**
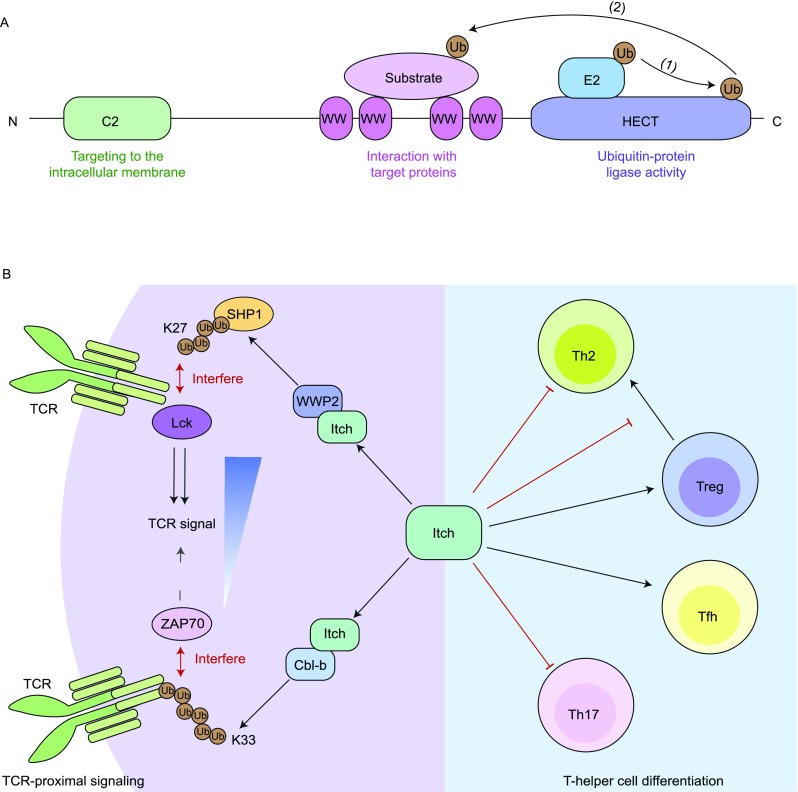
**Itch and its role in T cells**. (A) Schematic structure of Itch which shows the N-terminal C2 domain, the 4 WW domains for substrate recruitment, and the HECT ligase domain for the E2-ubiquitin binding and for the transfer of ubiquitin to the substrate. (B) Distinct functions of Itch in T cells. Itch cooperates with other E3 ligases to regulate TCR-proximal signaling (left). Itch regulates the differentiation of different T helper subsets (right)

The HECT-type family of E3 ligases possess an intrinsic catalytic activity that mediates protein ubiquitination and are crucial for immune cell development and functions (Aki et al., 2015). Itch is a HECT-domain containing E3 ligase whose structure includes an N-terminal C2 domain which binds to Ca^2+^ and phospholipids and functions in targeting to the intracellular membrane, four WW domains which mediate specific interactions with target proteins, and a C-terminal ubiquitin-protein ligase HECT domain (Fig. 2). Itch catalyzes protein ubiquitination in two-steps: (1) the HECT ligase domain binds to the E2-ubiquitin complex and transfers the activated ubiquitin to a catalytic cysteine residue at its C-terminal region; (2) Itch catalyzes the ubiquitin transfer from the HECT domain to the lysine residues of the substrates. Itch, which belongs to the neuronal precursor cell expressed developmentally downregulated protein 4 (NEDD4) family, has been demonstrated to regulate immune responses, especially Th cell-mediated adaptive immunity (Table 2). The biological function of Itch on immunological homeostasis was initially described in the genetic study of mouse coat color gene mutation in 1995 (Hustad et al., 1995). The mutant mice, who were homozygous for one of the identified mutations which ablated the HECT domain containing protein expression, exhibited various immunological disorders and skin scratching due to the constant itching; thus this responsible gene product was named Itch (Hustad et al., 1995; Perry et al., 1998).

**Table 2:** The major phenotype of Itch knockout mice.

Target cells	cKO mice	Phenotype	References
Th2 cell Inducible Treg cell Tfh cell Th17 cell	*Itch* ^*−/−*^	- increases IgE and IgG1 levels in the serum - CD4^+^ T cells produce more IL-4 and IL-5 - impaired inducible Treg generation - defective in Tfh differentiation - elevated IL-17 expression in the colonic mucosa	(Fang et al., 2002) (Venuprasad et al., 2006) (Venuprasad et al., 2008) (Xiao et al., 2014) (Kathania et al., 2016)
Treg cell	*Itch* ^fl/fl^ *Foxp3* ^*YFP-Cre*^	- increases secretion of Th2 cytokines in Treg cells - Th2-biased inflammation	(Jin et al., 2013)
Tfh cell	*Itch* ^fl/fl^ *Cd4-cre*	- defective in Tfh differentiation in a FOXO1-dependent manner	(Xiao et al., 2014),
Th2 cell	*Itch* ^fl/fl^ *Cd4-cre WWP2* ^*−/−*^	- spontaneous development of autoimmunity and lung inflammation - enhances Th2 differentiation - reduces TCR signal strength	(Aki et al., 2018)
CD4^+^ T cell	*Itch* ^*−/−*^ *Cblb* ^*−/−*^	- spontaneous autoimmunity - increases T cell signaling	(Huang et al., 2010)

### Itch in T helper cells

The multi-organ inflammation observed in Itchy (*Itch*^−/−^) mice was associated with a spontaneous Th2-skewed immune phenotype that showed higher levels of immunoglobulin E (IgE) and IgG1 in the serum compared to wild-type mice at 6 months of age (Fang et al., 2002). Indeed, Itch deficient CD4^+^ T cells produced more Th2 specific cytokines such as IL-4 and IL-5, suggesting that Itch is a negative regulator of Th2 differentiation (Fang et al., 2002; Venuprasad et al., 2006).

Further studies revealed the function of Itch in different types of Th cells. We found that naïve CD4^+^ T cells from Itchy mice show impaired inducible Treg generation cultured in the presence of TGF-β (Venuprasad et al., 2008). Moreover, the study using mice with Treg-specific ablation of Itch by crossing *Itch*^fl/fl^ mice to *Foxp3*^YFP-Cre^ mice demonstrated that Itch deficient Treg cells have normal suppressive function. However, secretion of Th2 cytokines is augmented in those cells, and *Itch*^fl/fl^ *Foxp3*^YFP-Cre^ mice show Th2-biased phenotypes (Jin et al., 2013). These findings suggest that Itch controls Th2 response by regulating Treg differentiation.

Follicular helper T (Tfh) cells are required for germinal center (GC) formation, affinity maturation of B cells, and generation of plasma and memory B cells. Itch deficiency results in impairment of Tfh cell differentiation and GC responses to acute viral infection in a T-cell intrinsic manner (Xiao et al., 2014). A mechanistic study demonstrated that Itch has a function in ubiquitin-mediated protein degradation of Foxo1, which negatively regulates Tfh development (Xiao et al., 2014; Stone et al., 2015). In addition, it is also reported that Itchy mice exhibit spontaneous colonic inflammation and increased colitis-associated colon cancer due to a higher expression of IL-17 in Th17 cells (Kathania et al., 2016). Itch might negatively control Th17 differentiation through ubiquitination mediated by lysine 48 (K48) linkage and degradation of RORγt, which is the master regulator of Th17 differentiation. Consistent with this, Nedd4 family interacting protein 1 (NDFIP1), which activates the catalytic activity of Itch, also promoted RORγt degradation and reduced IL-17 production in Th17 cells (Layman et al., 2017).

### Itch cooperation with other E3 ligases

Among NEDD4 E3s, Itch has a highly similar amino acid sequence to another member named WWP2, particularly in the WW domains that determine substrate recognition (Jiang et al., 2015; Aki et al., 2018). Recent structural and biochemical studies suggest that the molecular mechanisms by which catalytic activity of E3 is regulated are commonly employed between these two E3s (Riling et al., 2015; Chen et al., 2017; Zhu et al., 2017). Interestingly, it is demonstrated that some NEDD4 E3s recognize a common substrate and collaboratively function in cellular events via protein ubiquitination (Chaudhary and Maddika, 2014; Brigui et al., 2015; Chen et al., 2016). In spite of these findings, however, it has remained to be established whether Itch and WWP2 cooperate to regulate immune cells, especially Th cell development or function.

We recently generated *Itch*^*fl*/*fl*^ *Cd4*^*Cre*^ *WWP2*^−/−^ (DKO) mice with T cells that lack both Itch and WWP2 expression. DKO mice exhibit spontaneous development of autoimmunity and lung inflammation, to an extent not observed in any single deficient mice (Aki et al., 2018). DKO CD4^+^ T cells demonstrated hypo-responsiveness upon TCR stimulation and striking Th2 skewing. Mechanistically, Itch and WWP2 form a complex and cooperate to enhance TCR-proximal signaling, catalyzing K27-linked ubiquitin chain conjugation to SHP-1. Although Itch and WWP2 mediated ubiquitination of SHP-1 affects neither protein stability nor phosphatase activity of SHP-1, it is required for inhibition of its binding to Lck, thus positively regulating TCR-mediated signal transduction. These findings indicate the cooperative function of Itch and WWP2 on Th2 differentiation via regulating TCR signal strength.

Beyond the NEDD4 family, Itch has been shown to function together with a distinct E3 family in CD4^+^ T cells. Cbl-b, which belongs to the family of RING finger domain-containing E3s, is shown to be involved in the regulation of TCR-mediated T cell activation (Bachmaier et al., 2000; Chiang et al., 2000). Interestingly both Itch and Cbl-b are up-regulated during the induction of T cell anergy, and loss of Itch or Cbl-b in T cells results in resistance to anergy induction (Heissmeyer et al., 2004; Jeon et al., 2004; Venuprasad et al., 2006). These two E3s might target PLCγ1 for ubiquitination and subsequent degradation (Heissmeyer et al., 2004). These studies indicate the functional collaboration of Itch and Cbl-b on T cell anergy-mediated peripheral tolerance. Further study clearly showed that Itch and Cbl-b collaboratively prevent autoimmunity in mice by negative regulation of peripheral CD4^+^ T cell activation (Huang et al., 2010). Using a biochemical approach, we found that these two E3s interact with each other and cooperate to suppress proximal TCR signal transduction through promoting K33-linked polyubiquitination of the TCRζ chain in a proteolysis independent manner. Itch and Cbl-b mediated ubiquitination of the TCRζ chain inhibits the binding of TCRζ to ZAP-70 and phosphorylation of ZAP70, which leads to negative regulation of TCR signaling.

Although loss of either Itch or its functional partner E3, such as WWP2 or Cbl-b, in T cells had minimal to no overt effects on the spontaneous development of autoimmunity and inflammation in comparison with the combined loss of two E3s, it still remains elusive as to whether there is a functional compensation for Itch deficiency by another E3 ligase(s), in particular, the other NEDD4 E3s that share a structural homology with Itch. It is possible that such E3s could form a heteromeric complex with, and be potentially substituted for Itch or WWP2 in CD4^+^ T cell activation. A recently developed approach that is based on an E3-ubiquitin fusion protein that enables co-purification of Itch with potential interacting proteins will identify the protein complex formed *in vivo* containing E3s, and provide evidence on how such distinct E3s cooperatively regulate homeostasis in T cells via protein ubiquitination (O’Connor et al., 2015). In addition, future works will be required to elucidate the precise mechanism by which several types of ubiquitin conjugation such as K27-or K33-linkage in cooperation with two E3s could selectively occur and impact the assembly of signaling molecules including their target protein.

## Perspectives

Nearly two decades have passed since it was first identified that Cbl RING finger acts as a protein interacting domain to recruit an E2 ubiquitin-conjugating enzyme to promote transfer of the activated ubiquitin to itself or a substrate (Joazeiro et al., 1999). Significant progress has been made in many different aspects in the context of immune regulation. The availability of gene-targeted mice, particularly conditional knockout mice, has made it possible to dissect the roles of the E3 ligases in a particular immune cell type under specific experimental conditions. It is now understood that the same E3 ligase may function differently in different lymphocytes such as T cells vs. B cells, or different Th subsets such as Treg vs. Tfh cells. One explanation would be that the same E3 ligase may target different substrates in those cell types. It is also possible that these E3 ligases may exhibit unique activity by tagging the substrate in different ubiquitin linkages by using K27, K33, K48, or K63 residues. In cells, one E3 ligase may not work alone, but form a complex with other E3 ligases to exert their biological functions.

In the case of the VHL-HIF pathway, different biological functions have been reported in the same cell sub-type, such as in Tregs. Lymphoid tissues are constantly exposed to hypoxic challenges under steady and inflamed conditions. Even in the same local environments such as the germinal center, a gradient of oxygen levels has been observed (Abbott et al., 2016; Cho et al., 2016; Jellusova et al., 2017). It is possible that the experimental conditions employed may differ in those studies such as *in vitro* vs. *in vivo*, or steady state vs. after infection, which likely give rise to different biological readouts. More importantly, the metabolic states of those cells can be another factor that determines the final cell fate.

Either mutation or deletion of VHL or Itch has profound impact in human diseases such as tumor formation due to VHL mutation (Kim and Kaelin, 2004) or multisystem autoimmune disorders due to Itch deletion (Lohr et al., 2010), indicating the critical roles of the two E3 ligases in controlling human cell functions. It is still to be learned how the phenotypes in human diseases can be recapitulated in mouse models. In addition, the underlying cellular and molecular mechanisms are still far from clear to fully explain the observed biological manifestations in humans. These outstanding questions will lay a strong basis for future studies, and such knowledge will eventually provide help for the treatment of human diseases such as inflammation, autoimmune diseases, and cancer.
